# Meta-Analysis of Keratoconus Transcriptomic Data Revealed Altered RNA Editing Levels Impacting Keratin Genomic Clusters

**DOI:** 10.1167/iovs.64.7.12

**Published:** 2023-06-06

**Authors:** Enrico Bortoletto, Fabio Pieretti, Paola Brun, Paola Venier, Andrea Leonardi, Umberto Rosani

**Affiliations:** 1 Department of Biology, University of Padova, Padova, Italy; 2 Department of Molecular Medicine, Histology Unit, University of Padova, Padova, Italy; 3 Department of Neuroscience, Ophthalmology Unit, University of Padova, Padova, Italy

**Keywords:** RNA editing, keratoconus, adenosine deaminase acting on dsRNA (ADAR), recoding

## Abstract

**Introduction:**

Keratoconus (KC) is an ocular disorder with a multifactorial origin. Transcriptomic analyses (RNA-seq) revealed deregulations of coding (mRNA) and non-coding RNAs (ncRNAs) in KC, suggesting that mRNA-ncRNA co-regulations can promote the onset of KC. The present study investigates the modulation of RNA editing mediated by the adenosine deaminase acting on dsRNA (ADAR) enzyme in KC.

**Materials:**

The level of ADAR-mediated RNA editing in KC and healthy corneas were determined by two indexes in two different sequencing datasets. REDIportal was used to localize known editing sites, whereas new putative sites were de novo identified in the most extended dataset only and their possible impact was evaluated. Western Blot analysis was used to measure the level of ADAR1 in the cornea from independent samples.

**Results:**

KC was characterized by a statistically significant lower RNA-editing level compared to controls, resulting in a lower editing frequency, and less edited bases. The distribution of the editing sites along the human genome showed considerable differences between groups, particularly relevant in the chromosome 12 regions encoding for Keratin type II cluster. A total of 32 recoding sites were characterized, 17 representing novel sites. JUP, KRT17, KRT76, and KRT79 were edited with higher frequencies in KC than in controls, whereas BLCAP, COG3, KRT1, KRT75, and RRNAD1 were less edited. Both gene expression and protein levels of ADAR1 appeared not regulated between diseased and controls.

**Conclusions:**

Our findings demonstrated an altered RNA-editing in KC possibly linked to the peculiar cellular conditions. The functional implications should be further investigated.

Keratoconus (KC) is an asymmetric, usually bilateral, ectasia condition in which, as a result of thinning of the stroma, the cornea progressively assumes a conical shape potentially leading to a severe impairment in the vision quality.^1^^,^^2^ KC has its typical onset in adolescence and progresses until the third or fourth decade of life, being one of the most common reasons for cornea transplantation.^3^ Depending on the country, a variable incidence of KC has been reported (0.0003%-2.3%),^4^ with an estimation of 1.38 per 1000 (95% confidence interval [CI] = 1.14–1.62) based on 100 independent reports.^5^ KC etiology is likely multifactorial^2^^,^^6^ and although it is described as a noninflammatory disorder, a strong association with allergic eye disease, eye rubbing, and repeated trauma due to contact lenses has been reported.^7^^–^^10^

RNA expression analyses of KC corneas showed increased levels of several cytokines, like IL-1α, IL-1β, and TNF-α.^11^^,^^12^ In addition IL-1α and TNF receptors are highly expressed in fibroblasts obtained from patients with KC.^11^^,^^13^ Both IL-1α and IL-1β induce apoptosis in corneal endothelium synergistically with cytokines, such as TNF-α, via the production of reactive nitrogen species.^14^ Interestingly, exclusively in KC, IL-1α contributes to corneal oxidative damage, where it was shown to specifically downregulate the level of extracellular-superoxide dismutase (SOD3), in cultured KC stromal cells.^15^ The higher expression of the short form of the Sp3 transcription factor in KC decreased the expression levels of TrkANGFR, nerve growth factor (NGF) and p75NTR, compared to healthy corneas.^16^ Moreover, keratin5 (KRT5), KRT12, KRT14, and KRT16 were reported to be overexpressed in patients with KC.^17^ Among all the different keratin proteins, the corneal epithelium's differentiation-related and cell type-specific keratin pair is KRT3 (type II)/KRT12 (type I). However, the possible role of the keratin proteins has not been described so far.^18^^,^^19^

Genomewide association studies of central corneal thickness and KC, suggest that variation associated with *VSX1*, *LOX*, *ZNF469*, *SOD1*, *TGFBI*, *FOXO1*, *FNDC3B*, *ZFN469*, *COL5A1*, and *AKAP13* can be implicated in KC disease.^20^^–^^22^ Overall, although different genes are potentially involved in the KC etiology, their mechanistic role is still unclear. Together with gene expression alterations, modifications occurring on RNA molecules (epitranscriptomic) can also influence cellular functioning, as showed for the nervous system development, or contribute to proteome diversification and regulation of the immune system.^23^ A number of epitranscriptomic modifications, including alterations in the base composition of RNA molecules, isomerization of uridine to pseudouridine, methylation of the ribose 2′-hydroxyl group among 160 known modifications, have been reported, and their deregulation has been often associated with pathological conditions in humans and in other model species.^23^^–^^25^ Although the identification of modified RNA nucleotides is challenging,^26^ the deamination of adenosine to inosine mediated by the enzyme adenosine deaminase acting on dsRNA (ADAR) can be detected tracing multiple A-to-G mismatches (hyper-editing) occurring along RNA molecules.^26^ This process, known as A-to-I editing and impacting RNA molecules forming double-strand (ds) structures, has been studied in physiology and in association with cancer and autoimmune diseases.^27^^–^^30^

Although ADAR-mediated variations can also impact coding (mRNA) and non-coding RNAs (ncRNAs), most of the editing events occurred on interspersed genomic repeats.^28^^–^^32^ Alterations in the RNA editing levels have been correlated with disorders of the nervous system, such as amyotrophic lateral sclerosis,^33^ epilepsy,^34^ schizophrenia and bipolar disorder,^35^ and autism,^36^ with the report of a reduced activity of editing compared to controls for most of these diseases.^37^ Additionally, ADAR1-mediated editing can limit the activation of innate immune defenses upon sensing of endogenous dsRNAs.^38^ Taken together, ADAR editing is essential for the maintenance of organism homeostasis^39^^,^^40^ and altered editing can cause a rise of nonsynonymous mutations (called recoding events), can create editing-assisted splicing events, or modify the targets of miRNAs, with paramount implication for cellular functioning.^32^

Additionally, ADAR1 activity plays a role in several processes, including the structural modification of the protein, viral inactivation, and subsequent cell survival. However, its function in the skin and role in the development in skin related diseases, such as Dyschromatosis symmetrica hereditaria, is still unknown. Finally, epitranscriptomic modifications have never been investigated in association with ocular diseases, nor the possible activity of ADAR. Therefore, we conducted a meta-analysis of two transcriptomic datasets including KC and control samples to evaluate perturbation in the level of ADAR-mediated RNA editing. We further considered the distribution of the editing sites along the human genome, the presence of differentially edited genes, and of recoding sites.

## Materials and Methods

### Data Retrieving

The human genome and related gene annotations were retrieved from the USCS genome browser^41^ (ID: GRCh37, hg19)*.* RNA sequencing (RNA-seq) datasets of patients with KC and matched controls (CTs) were downloaded from the NCBI SRA archive under the project ID PRJNA636666 and PRJNA312169. Kwon editing sites were retrieved from the REDIportal database.^42^ The patients’ metadata information associated with this experiment can be found in the original papers.^43^^,^^44^

### Preliminary Data Analysis

Raw RNA-seq reads were trimmed for quality using Trimmomatic^45^ adopting the following parameters: LEADING:3 TRAILING:3 SLIDINGWINDOW:4:15 MINLEN:50. Trimmed reads were mapped to the human reference genome using STAR version 2.6.0,^46^ with the following parameters –outFilterMultimapNmax 1 –alignSJoverhangMin 8 –alignSJDBoverhangMin 1 –outFilterMismatchNmax 999 –outFilterMismatchNoverLmax 0.04 –alignIntronMin 20 –alignIntronMax 1000000 –alignMatesGapMax 1000000. All the format conversions and the managing of mapping files were performed with SAMtools.^47^

### ADAR Hyper-Editing Analysis

The *hyperediting* tool^48^ was applied after minimal modifications required to overcome software incompatibilities of the original version. The parameters were adapted applying: 5 for Minimum of edited sites at Ultra-Edit read (%); 60 for Minimum fraction of edit sites/mismatched sites (%); 25 for Minimum sequence quality for counting editing event (PHRED); 60 for Maximum fraction of same letter in cluster (%); 20 Minimum of cluster length (%); and imposing that the hyper-editing clusters should not be completely included in the first or last 20% of the read. The identified reads were realigned with *pblat*^49^ to the reference genome in order to exclude possible miss-mapping and the final subsets of hyper-edited reads were mapped on the reference genome and the occurrences per gene were counted using CLC Genomic Workbench version 22 (Qiagen, US).

### Detection of Editing Sites

The workflow used in this work is described in ref.^50^, except for some modifications described below. Editing events were detected de novo using *REDIToolsDenovo.py.*^51^ Sites with multi-mismatches per single position and sites in homo-polymorphic regions were excluded. Known single nucleotide polymorphisms (SNPs), repeated elements, and known editing events stored in the UCSC genome browser,^41^ and the REDIportal^42^ databases, respectively, were annotated on the reference genome. The putative ADAR-editing sites were then divided into three groups according to their overlapping annotation: ALU repeats (ALU), other type of repeats (REP_NON_ALU), and non-repetitive region (NON_REP). NON_REP and REP_NON_ALU sites undergo more stringent call criteria than ALU sites that consider mis-mapping reads and PCR duplicates. Filtered positions were considered RNA editing candidates.

### Computation of Editing Indexes

Two different editing indexes were calculated for the 26 RNA-seq datasets.

(1) The ALU editing index, namely the ratio of A-to-G mismatches divided by the total coverage of all “As” within Alu elements and multiplied by 100, was calculated as previously indicated using *RNAEditingIndexer.*^52^ This index provided a normalized measure of editing activity, useful to compare different tissues and experiments.
(2) The overall RNA editing level was calculated per sample using the *getOverallEditing.py* script obtained from QEdit (https://github.com/BioinfoUNIBA/QEdit). The overall editing index is defined as the number of reads with G were in the genome, there is an A at all known editing positions over the number of all reads covering the positions without imposing specific sequencing coverage criteria.^53^
(3) The hyper-editing levels was calculated dividing the number of hyper-edited reads by the total mapped reads on the genome and multiplying per 1000. Genes were considered “hyper-edited” if they are retrieved in at least 70% of the samples belonging to a given group.

### Gene Expression Analysis and Hyper-Editing Normalization

To identify differentially expressed genes (DEGs), the trimmed reads were mapped on the human reference genome (hg19) applying the following parameters: Mismatch cost = 2; Insertion cost = 3; Deletion cost = 3; Length fraction = 0.8; Similarity fraction = 0.8, and expression values were counted as Read Per Kilobase of Mapped reads (RPKM). A Baggerley test with false discovery rate (FDR) *P* value correction was applied to identify DEGs, setting a cutoff of 2-fold changes (FCs) and a 0.01 of FDR-corrected *P* value. A GO-enrichment analysis based on Uniprot gene ontology (goa_human_20200423_hg19) was performed, removing hits with mean RPKM below 5.0, absolute fold change below 2 and with an FDR *P* value higher than 0.01. To normalize hyper-editing levels, gene expression values were computed also as number of uniquely mapped reads.

### ADAR1 Protein Level Determination by Western Blot Analysis

Three corneas from patients with KC (3 men, mean age = 36 ± 5 years) at stage 3 who underwent penetrating keratoplasty and 3 corneas from healthy donors (3 men, mean age = 53 ± 12 years) obtained at the Fondazione Banca degli Occhi del Veneto (Venice, Italy), were included in the study. A written informed consent was obtained from all the patients with KC before obtaining tissue specimens. This research study was approved by the Institutional Review Board and Local Ethical Committee and adhered to the tenets of the Declaration of Helsinki. By Italian law, written informed consent from donor's next of kin was obtained for the use of tissues for transplantation or, as an alternative, for research purposes in agreement with the Declaration of Helsinki. The tissues were used in accordance with the laws of the National Transplant Center (Rome. Italy). Corneas were maintained in storage medium at 31°C until sample collection.

Corneal tissues from control and KC subjects were lysed in RIPA lysis buffer (sodium deoxycholate 0.5%, Triton 1%, EDTA 10 mM in PBS buffer) plus proteases inhibitors cocktail (Thermo Fisher Scientific, Waltham, MA, USA) and stored at -80°C until use. Protein concentration was measured using a BCA protein assay kit (Merck, Germany). For Western blotting analysis, proteins were added to the sample loading buffer (62.5 mM Tris pH 6.8, 10% v/v glycerol, 2% w/v sodium dodecyl sulphate, 5% v/v β-mercaptoethanol, and 0.1% w/v bromophenol blue), denatured at 70°C for 5 minutes and separated by 10% sodium dodecyl sulphate-polyacrylamide gel electrophoresis (SDS-PAGE). Proteins were transferred to PVDF membrane for 1 hour at 4°C with the constant current of 100 V, in blotting buffer (25 mM Tris, 192 mM Glycine, and 20% Methanol). Non-specific binding sites were blocked incubating the membrane for 1 hour at 25°C in 5% w/v non-fat dry milk in 20 mM Tris (pH 7.6), Tween (Sigma Aldrich, St. Louis, MO, USA) 0,05%, 150 mM NaCl (Tris-Buffered Saline [TBST]). The membrane was then incubated overnight at 4°C with rabbit anti human-ADAR1 (PA5-21369, Thermo Fisher) diluted 1:1000 antibody in TBST. After 3 washes for 10 minutes in TBST, the membrane was incubated with anti-rabbit HRP (Horseradish Peroxidase)-conjugated antibody (Cell Signaling Technology) diluted in TBST 1:2000, for 1 hour at 25°C. The antibody reaction was revealed by chemiluminescence using LiteUp WB chemiluminescent Substrate (Euroclone, Italy) and ADAR1 presence was revealed by ImageQuant LAS 4000 (GE Healthcare, Chicago, IL, USA). The blots were sequentially stripped and incubated at 25°C for 1 hour with primary monoclonal mouse anti-GADPH antibody (Merck) 1:2000 and processed as described above. ImageJ software was used to obtain the relative quantity of ADAR1 in the samples and densitometry values were normalized with the ones of GADPH.

### Statistical Analysis

Because the data were not normally distributed (Shapiro-Wilk test), we used the Kruskal-Wallis test and the Mann-Whitney *U* test for analyzing the specific sample pairs for stochastic dominance (nonparametric statistical test), using Bonferroni correction of the *P* value. The z-test was performed with the BSDA package. The representation of chromosome 12 was performed with the Gviz package.^54^ Western blotting was performed in triplicate and the comparison of protein levels between corneal tissues was analyzed with the 1-way ANOVA test. For the statistical significance, differences were indicated at *P* < 0.05 (*). All the statistical analyses were performed using R software version 4.1.2^55^ and GraphPad Prisma 9 (San Diego, CA, USA).

## Results

### ADAR-Mediated Editing is Lower in Keratoconus Samples

**Figure 1.**
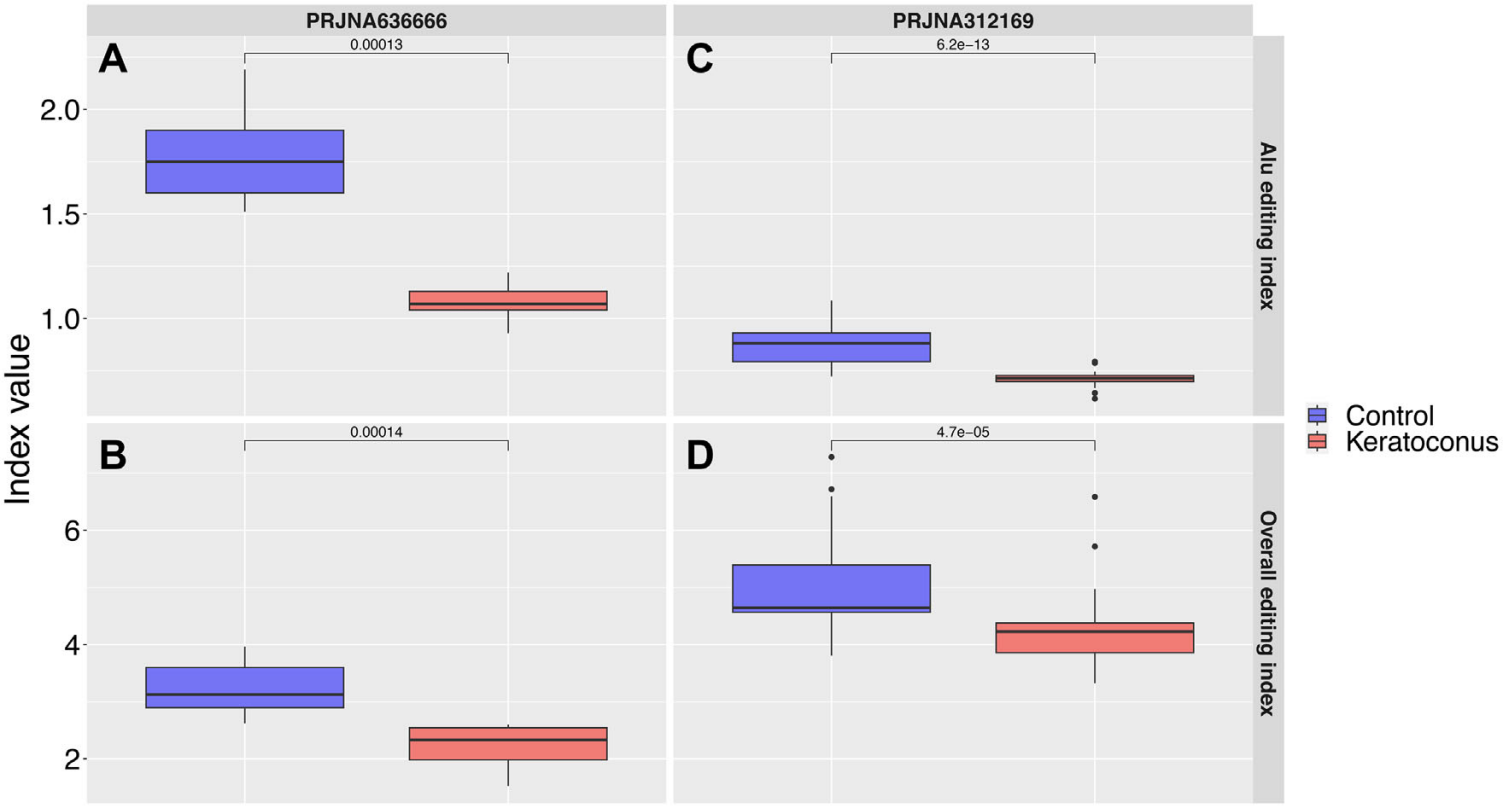
(**A**) The box plots showed the value of the ALU editing index and overall editing index and the hyper-editing level for Control (*blue*) or keratoconus (*red*) samples for EXP1 (**A, C**) and EXP2 (**B, D**).

**Figure 2.**
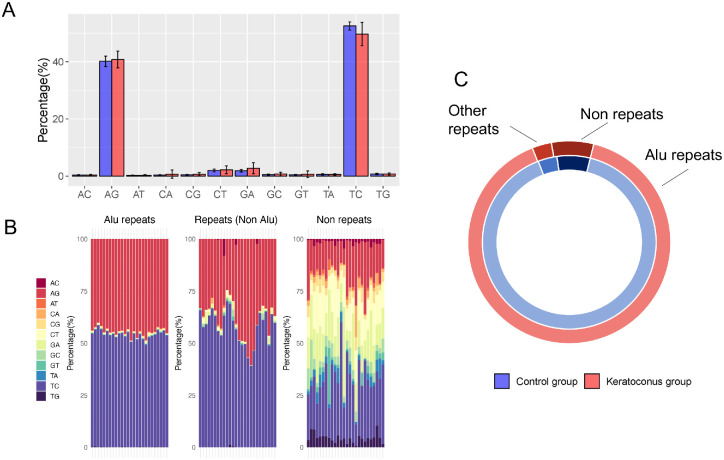
(**A**) The histogram depicted the average abundance of each variation type in control or keratoconus groups (*blue* and *red*, respectively). (**B**) Relative abundance of the different variations in each of the 26 samples, divided by ALU repeats, other repeats (non-Alu) and non-repetitive regions (coding). (**C**) Relative distribution of ADAR compatible changes among ALU repeats, other repeats and non-repeated regions (coding) for control (*blue*) and keratoconus (*red*) samples.

The ALU editing index computed on the control corneas revealed editing levels comparable with human brain regions (1.78 ± 0.24%; Supplementary Fig. S1). We then computed 2 editing indexes to compare the editing levels in KC versus CT samples in experiment 1 (EXP1, *N* = 26) and experiment 2 (EXP2, *N* = 20). KC samples showed a mean ALU editing index of 1.08 ± 0.08, compared to 1.78 ± 0.24 for the CT group in EXP1 (Fig. 1A, *P* = 0.00013, Mann-Whitney *U* test), a reduction confirmed also in EXP2, with 0.71 ± 0.04 in the KC group compared to 0.88 ± 0.1 in the CT group (*P* = 6.2e^−13^, Mann-Whitney *U* test; Fig. 1B). The overall editing index also supported the reduced RNA editing in the KC group, with 4.3 ± 0.66 in the KC group compared to 5.08 ± 0.0.88 in the CT group for Exp1 (*P* = 4.7e^−05^, Mann-Whitney *U* test; Fig. 1C) and 2.26 ± 0.32 in the KC group compared to 3.24 ± 0.53 in the CT group for EXP2 (*P* = 0.00014, Mann-Whitney *U* test, Fig. 1D)*.* The frequencies of the different nucleotide variations revealed that most of the identified editing events are compatible with the activity of ADAR enzymes, resulting in 91.5 ± 5.05% of A-to-G and T-to-C variations, with no difference comparing KC versus CT samples (Fig. 2A, Supplementary Fig. S2). Notably, most of the variations not compatible with ADAR impacted non-repeated regions were C-to-T and G-to-A variations (Fig. 2B).

### Genomic Distribution of the RNA-Editing Sites

The great majority (90%) of editing sites are located among ALU repeats, a small fraction localized on other interspersed repeats (4% and 3% for KC and CT, respectively), whereas 6% and 7% impacted non-repetitive genomic regions (Fig. 2C). A total of 53,363 editing sites were detected in the CT group (44,651 in ALU repeats, 3395 in other repeats, and 5317 in non-repetitive regions) and 39,835 editing sites were detected in the KC group (34,634 in ALU repeats, 2698 in other repeats, and 2503 in non-repetitive regions). The circos plot depicting the distribution of these editing event in the different human chromosome considering the showed a denser distribution in chr15, chr16, chr17, and chr19, compared to chr13, chrX, or chrY (Supplementary Fig. S3).

**Figure 3.**
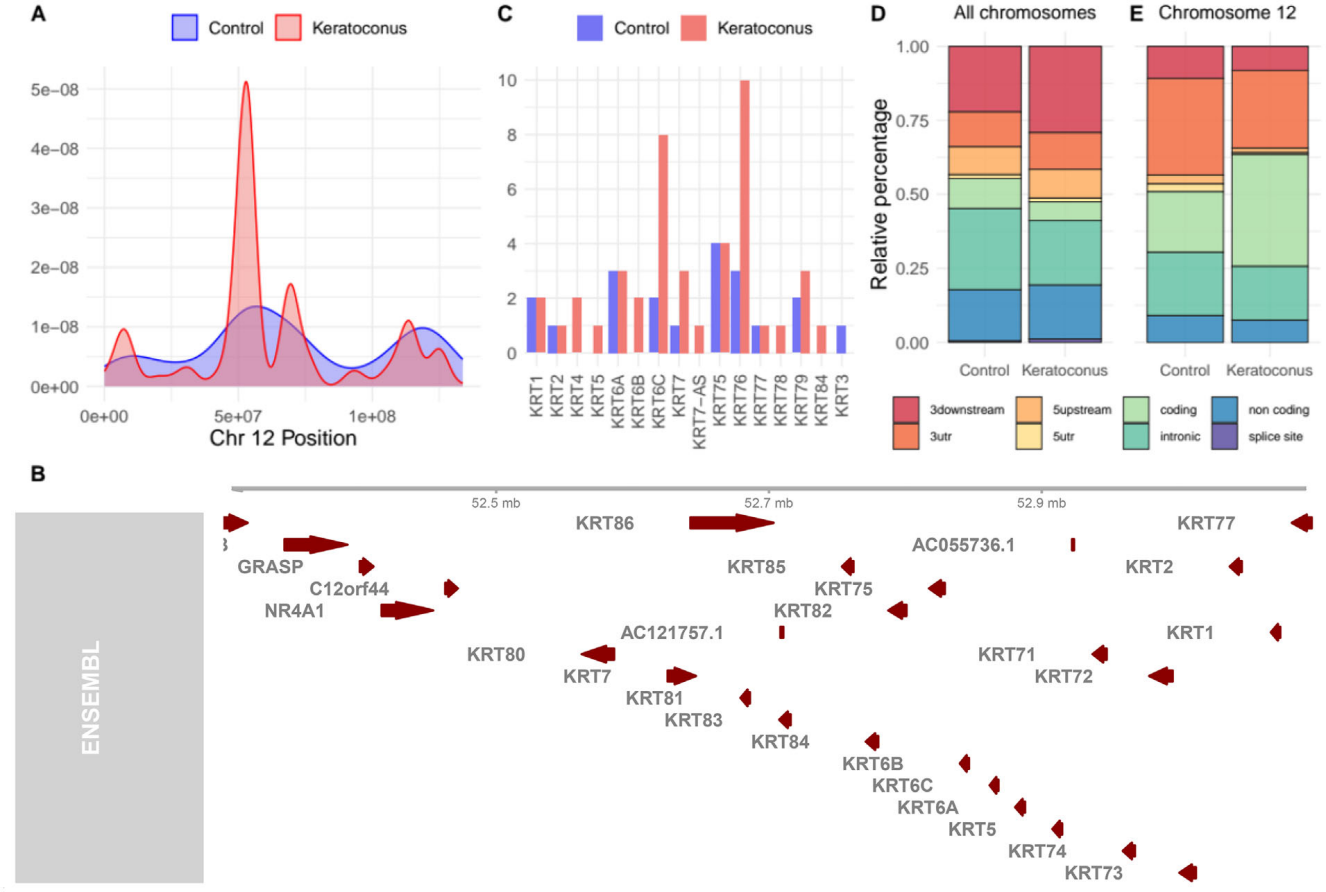
(**A**) Density plot representing the distribution of the editing sites along the chromosome 12. (**B**) The picture depicts the genes included in the point where the editing is most condensed in the keratoconus group, all the *red arrows* represent a coding gene. (**C**) The histogram shows the number of different editing site position retrieved for each keratin genes included in the keratin type II cluster. The stacked bar plot represents the relative distribution of the editing sites in 3′ downstream, 3′ UTR, coding region, splicing sites, 5′ upstream, 5′ UTR, non-coding RNAs, and intronic region comparing the profiles of the control and keratoconus group considering all chromosomes (**D**) and only chromosome 12 (**E**). In all the different panels, the keratoconus group is represented with the color *red* and the control group with the color *blue*.

To compare the distribution of the editing events along the chromosomes between the KC and CT groups, a density plot was created, and significative distribution differences identified with a z-test, resulting significative for all the chromosomes (Supplementary Fig. S4). Chromosome 12 resulted to be the one with the most unbalanced distribution, with a *P* value of 2.2 e^−16^ (Fig. 3A). For the KC group only, an editing peak was evident in the genomic region between 42 to 61 Mb, with the maximus between 52 to 53 Mb, a region encoding 24 genes, including a cluster of keratin type II (Fig. 3B). Accordingly, we investigate the number of editing sites that impacted the keratin type II cluster between KC and CT (Fig. 3C), resulting similarly for KRT1, KRT2, KRT6A, KRT75, and KRT77, but higher in KC for KRT4, KRT5, KRT6B, KRT6C, KRT7, KRT7-AS, KRT76, KRT78, KRT79, and KRT84, whereas it was lower in KC only for KRT3. Among all the listed keratins only KRT1, KRT4, KRT76, and KRT78 are upregulated in keratoconus all the other keratins are not differentially expressed (Supplementary Table S1). Considering the distribution of the editing sites between 3′ downstream, 3′ UTR, coding region, splicing sites, 5′ upstream, 5′ UTR, non-coding RNAs, and intronic regions we could show that considering all the chromosomes the editing profile differs between the CT and KC groups for a higher amount of editing sites in intronic regions and a lower amount in 3′ downstream (Fig. 3D). Differently, considering the chromosome 12 only, the editing sites in the KC group are more concentrated in the coding regions and less in the 3′ UTR (Fig. 3E).

### Alteration in the Hyper Editing Activity of ADAR1

**Figure 4.**
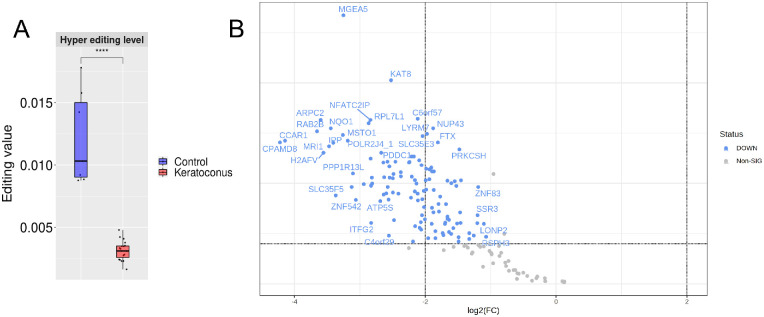
(**A**) The box plot shows the hyper-editing level, calculated dividing the number of hyper-edited reads by the total mapped reads on the genome and multiplying per 1000, in the keratoconus group (*red*) and the control group (*blue*). (**B**) Volcano plot comparing the hyper editing frequency among the genes hyper edited in both the experimental groups.

The ADAR1-medited hyper editing activity estimated with the hyper editing level (see Materials and Methods section) resulted significantly lower in the KC group compared to the CT group (0.003 ± 0.0007‰ vs. 0.012 ± 0.003‰, *P* = 0.0001, Mann-Whitney *U* test; Fig. 4A). To investigate if the reduced levels of ADAR editing in KC originated from lower editing frequencies or from less edited bases, we evaluated gene-specific hyper-editing levels. As a result, 19 genes appeared hyper-edited in KC only, 994 genes in CT only, and 163 genes in both groups. A total of 121 out of these 163 genes were differentially hyper-edited, most of them with higher hyper-editing levels in CT (FDR *P* value threshold 0.01; Fig. 4B). The average number of hyper-edited genes per samples is significantly higher in the CT group, with 2,558 ± 619 hyper-edited genes compared to 831 ±1 91 in the KC group (*P* = 0.0001, Mann-Whitney *U* test; Supplementary Fig. S5).

### Impact of RNA Editing Within Whole Gene Expression Profiles

Gene expression analysis of KC versus CT samples revealed 1263 DEGs, mostly resulting downregulated (78%). Intriguingly, the expression of both KRT3 and KRT12 increase in the KC group, from 2244 to 5995 RPKMs and from 11,908 to 21,753 RPKMs, respectively. Gene Ontology Biological Pathways (GOBP) revealed 55 dysregulated terms comprising 549 genes, 80% of them downregulated in KC. Editing enzymes of the APOBEC family appeared also downregulated (APOBEC3A and APOBEC3B, fold change of −12.3 and −10.0, respectively), such as several genes involved in the interferon pathway (e.g. SOCS3, IFI6, IRF6, and ISG15 among others; see Supplementary Table S1). Notably, the expression of ADAR1 was slightly reduced in the CT group, but this variation is not statistically significant (14 vs. 25 RPKMs in CT and KC, respectively).

Considering KC and CT groups, a total of 32 recoding sites were identified, 15 already known in REDIportal and 17 identified as new putative editing sites (see the Table). Nine of the novel editing sites appeared differentially edited between the two conditions, with JUP, KRT17, KRT76, and KRT79 edited with higher frequencies in the KC group, whereas BLCAP, COG3, KRT1, KRT75, and RRNAD1 edited with lower frequencies. No correlation was observed between editing frequency and expression levels of these genes (Supplementary Table S2). Genes with stable expression levels but differentially impacted by editing were also observed, like RRNAD1 (FC of 1.48), which is edited in all the CT samples but only in one KC sample; COG3 (FC of 1.09), edited in the 70% of CT samples but only in 15% of KC ones and JUP (FC of −1.62), edited in 57% of KT samples and only in 14% of CT ones. Among others, AZIN1, CCNI, CLDN3, and HLA-G appeared edited only in the CT group (see the Table), although AZIN1 and CLDN3 are expressed also in KC samples (see Supplementary Table S1).

**Table.** List of the Non-Synonymous Mutations Supported by at Least 10 RNA Reads and Commonly Found in the Two Groups

Gene	Protein Name	Protein	AA Change	Freq C	Freq. K
ENSG00000166619	Bladder cancer-associated protein	BLCAP	Q5R	0.15	0.10
ENSG00000166619	Bladder cancer-associated protein	BLCAP	Y2C	0.21	0.13
ENSG00000065883	Cyclin-dependent kinase 13	CDK13	Q103R	0.17	1.00
ENSG00000065883	Cyclin-dependent kinase 13	CDK13	Q35R	0.58	0.25
ENSG00000136152	Conserved oligomeric Golgi complex subunit 3	COG3	I635V	0.29	0.20
ENSG00000122218	Coatomer subunit alpha	COPA	I164V	0.17	0.19
ENSG00000227184	Epiplakin	EPPK1	E1744G	0.36	0.16
ENSG00000196924	Filamin-A	FLNA	Q2341R	0.10	0.19
ENSG00000136068	Filamin-B	FLNB	M2324V	0.20	0.12
ENSG00000204632	HLA class I histocompatibility antigen, alpha chain G	HLA-G	H193R	0.77	0.93
ENSG00000204389	Heat shock 70 kDa protein 1A	HSPA1A	I119T	0.19	0.19
ENSG00000163453	Insulin-like growth factor-binding protein 7	IGFBP7	K95R	0.13	0.13
ENSG00000128422	Keratin, type I cytoskeletal 17	KRT17	I237V	0.10	0.50
ENSG00000167768	Keratin, type II cytoskeletal 1	KRT1	K257R	0.98	0.71
ENSG00000173801	Junction plakoglobin	JUP	I396V	0.10	0.50
ENSG00000170454	Keratin, type II cytoskeletal 75	KRT75	I440T	0.66	0.96
ENSG00000185069	Keratin, type II cytoskeletal 2 oral	KRT76	S63G	0.44	0.84
ENSG00000189182	Keratin, type II cytoskeletal 1b	KRT77	I320V	0.95	1.00
ENSG00000185640	Keratin, type II cytoskeletal 79	KRT79	T423A	1.00	0.97
ENSG00000185640	Keratin, type II cytoskeletal 79	KRT79	V435A	0.35	0.34
ENSG00000181143	Mucin-16	MUC16	H12407R	0.17	0.15
ENSG00000181143	Mucin-16	MUC16	S12687P	0.30	0.19
ENSG00000219481	Neuroblastoma breakpoint family member 1	NBPF1	D959G	0.29	0.31
ENSG00000219481	Neuroblastoma breakpoint family member 1	NBPF1	S961P	0.34	0.37
ENSG00000140398	Endonuclease 8-like 1	NEIL1	K242R	0.95	0.87
ENSG00000185290	Nuclear protein, transcriptional regulator, 1-like	NUPR1L	K91R	0.75	0.60
ENSG00000146733	Phosphoserine phosphatase	PSPH	L223P	0.65	0.38
ENSG00000188846	60S ribosomal protein L14	RPL14	K147E	0.28	0.19
ENSG00000143303	Protein RRNAD1	RRNAD1	Q247R	0.25	0.11
ENSG00000087266	SH3 domain-binding protein 2	SH3BP2	R561G	0.16	0.10
ENSG00000159140	Protein SON	SON	R580G	0.16	0.10
ENSG00000204514	Zinc finger protein 814	ZNF814	Y744H	0.25	0.10
ENSG00000155096	Antizyme Inhibitor 1	AZIN1	S367G	0.16	–
ENSG00000118816	Cyclin I	CCNI	R75G	0.10	–
ENSG00000165215	Claudin 3	CLDN3	I40V	0.31	–
ENSG00000204632	Major histocompatibility complex, class I, G	HLA-G	Y221H	0.25	–

Underlined variations are the ones differentially edited between the two conditions. At the bottom of the table, group-specific variations discussed in the main text have been also reported.

### Western Blot Analysis of ADAR1 Revealed Stable Protein Levels

**Figure 5.**
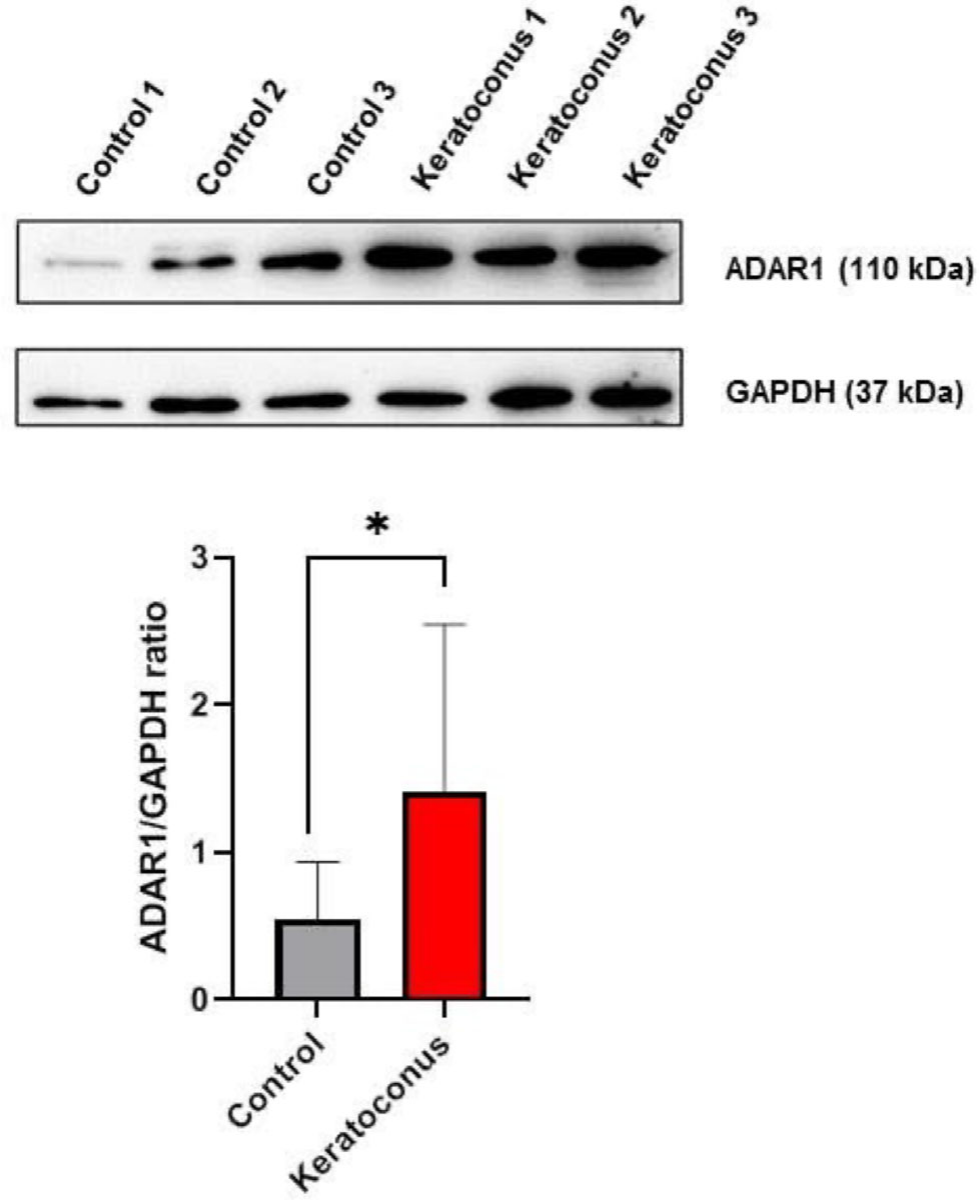
Western Blot analysis of keratoconus and control corneas (average of 3 replicates is reported). (**A**) Blotting images of one representative experiment. (**B**) ADAR1/GDPH ratio were significantly increased in keratoconus biopsies (* = *P* < 0.05).

Western Blot analysis of three independent KC and CT cornea samples suggested an increase of ADAR1 protein level in KC (2.5 folds, *P* < 0.05), although differences between the different samples (Fig. 5) were observed.

## Discussion

Whole transcriptome analysis of KC samples revealed deregulations of several genes compared to normal corneas, including ncRNAs^56^ and miRNAs.^57^ Despite that expression data are available for several genes, a comprehensive model describing the transcriptional modulations modifications in KC is missing and epitranscriptomic modifications have never been considered. Notably, genetic-encoded C-to-U variation occurring on the seed region of *mir-184* has been previously associated with KC,^58^ suggesting that single nucleotide modifications can have an important impact also on this disease.

By meta-analysis of two whole transcriptome datasets, we have traced RNA modifications in in KC corneas and, after comparing them with CT corneas, we could reveal a marked reduction of RNA editing and hyper-editing levels in KC samples. This reduction both impacts the overall editing frequencies and the total number of edited bases. Most of RNA editing events are directed toward genomic repeats, particularly ALU elements, as previously reported,^59^ with a clear predominance of ADAR-compatible edits, in the form of A-to-G and C-to-T variations. Conversely, the part of editing impacting non-repeated genomic regions (including coding genes) revealed a considerable fraction of C-to-T and G-to-A variations, which can be possibly mediated by other RNA editing enzymes, such as members of the APOBEC family.^60^ In our analysis, APOBEC3A and APOBEC3B appeared downregulated in KC samples, possibly suggesting a connection with the reduced editing in these samples. Differently from APOBECs, the expression of ADAR1 resulted slightly upregulated in KC samples considering RNA-seq samples, a result also suggested by Western Blot analysis performed on three independent KC and CT corneas. Despite this slight increase of mRNA and protein levels of ADAR1, the editing activity appeared strongly reduced. Unexpectedly, we did not observe an increase of expression of antiviral genes, which have been reported to be maintained under control by ADAR hyper-editing.^38^ On the contrary, the expression of these genes appeared downregulated.

Notably, most of the editing sites in patients with KC are concentrated in the keratin type I and type II clusters. Several of the keratins displaying higher number of editing sites in the KC group have been reported as involved in different human diseases, such as KRT4 in White sponge nevus, KRT5 in epidermolytic hyperkeratosis, KRT6C in focal or diffuse nonepidermolytic palmoplantar keratoderma and KRT76 in plantar wart.^61^^–^^63^ However, the functional e role of keratins in KC has not been deeply investigated. KRT3/KRT12 is a specific pair of paired 55/64 kDa keratins that are expressed by corneal epithelial cells. KRT12 seems to be helpful as a marker for separating central corneal cells from limbal stem cells, and the KRT3 may play a role in inflammatory eye disease.^64^ Because KRT12 mutant mice are more susceptible to recurrent erosion (epithelial cell loss), this protein may play a crucial role in corneal epithelial connections.^65^ Notably, even if both KRT3 and KRT12 are more expressed in the KC group, they show fewer editing sites.

Interestingly, beside the roles played by ADAR hyper-editing, site-specific edits can have crucial functional impacts.^66^^,^^67^ Among the limited number of recoding events, we highlighted potential interesting targets, like plakoglobin (JUP) which is more edited in KC samples. Loss-of-function mutation of JUP has been associated with Naxos disease,^68^ which is also characterized by palmoplantar keratoderma. The higher editing levels observed for three keratin RNAs (KRT17, KRT76, and KRT79) could be associated with a loss of organization of these proteins, typical of skin disease.^69^ Differently, other genes with reduced recoding levels in KC can loss their function. A pertinent example is the recoding site of COG3 (I635V), conserved through the evolution of vertebrate and possibly exerting a functional role.^70^ COG3 is part of the COG complex required for normal Golgi morphology and localization.^71^ Another gene with reduced recoding events is RRNAD1 (METTL25B), which is part of the methyltransferase-like gene family, also involved in the conversion of adenosine to m6A in pri-miRNAs, and this variation can have a deep impact on seed-target recognition of miRNAs.^72^ Finally, AZIN1 (antizyme inhibitor 1), is edited on the S367G position only in control samples. This recoding event, often associated with COG3 editing as in our case, provide an increase in protein stability, and a stronger affinity to antizyme, thereby facilitating entry into cell cycle, that in the case of cancer increase the malignancy.^73^

## Conclusions and Future Research Directions

The heterogenicity of RNA-seq data and the absence of paired genomic data reduced the power of detecting editing sites, and we considered them as a limitation of our analysis. In the absence of paired DNA- and RNA sequencing data, alternative approaches to directly detect modified nucleotide (inosines in our case) from native RNA should be pursued. Together with approaches aiming to functionally characterize the impact of ADAR editing, such as proteomics, the determination of native RNA modification in KC and normal corneas will challenge future research as they can shed a functional light into the epitranscriptome. The multiple reports of transcriptional down-regulations in KC^44^^,^^56^ suggested a systematic downshift of gene expression, whose mechanism should be elucidated and for which ADAR editing can play a regulative role.

## Supplementary Material

Supplement 1

Supplement 2

Supplement 3

Supplement 4

Supplement 5

Supplement 6

Supplement 7
