# Supplementary figures and images for: Meta-Analysis of Keratoconus Transcriptomic Data Revealed Altered RNA Editing Levels Impacting Keratin Genomic Clusters
Source: Invest Ophthalmol Vis Sci. 2023 Jun 6;64(7):12. doi: 10.1167/iovs.64.7.12 (PMC10249681; doi:10.1167/iovs.64.7.12)

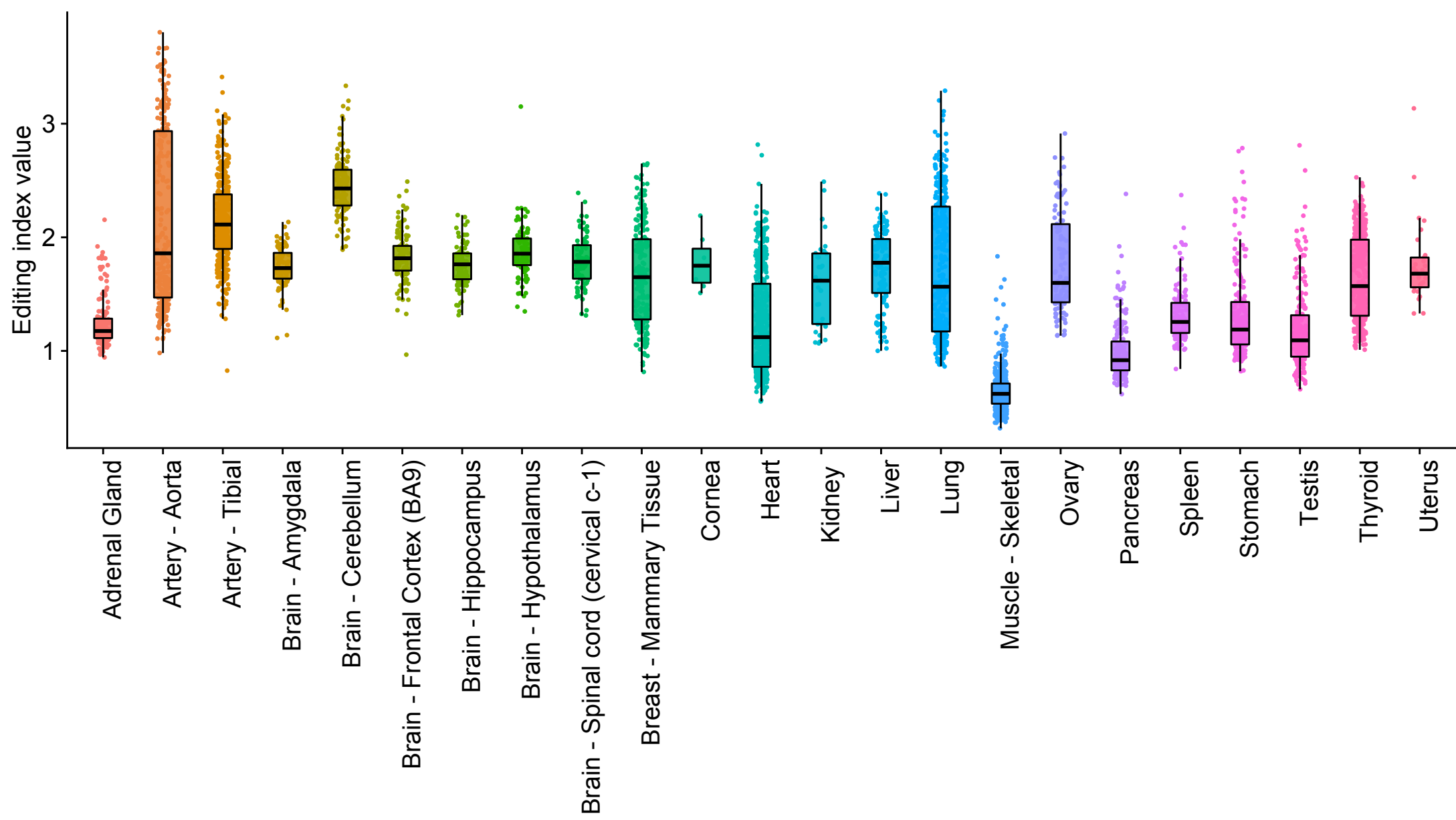

Supplement: Supplement 1 [file iovs-64-7-12_s001.pdf]

# Control group

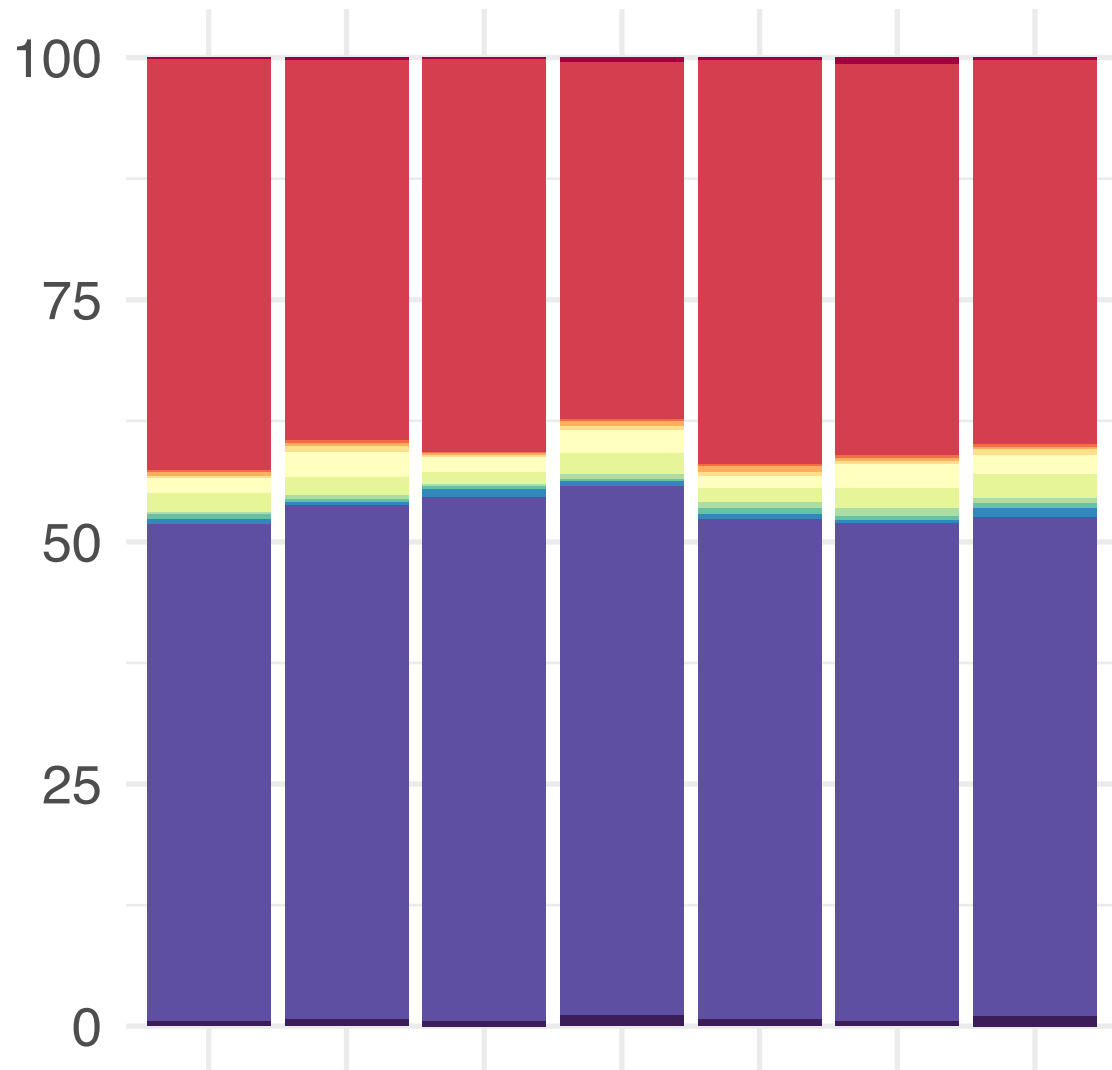

# Keratoconus group

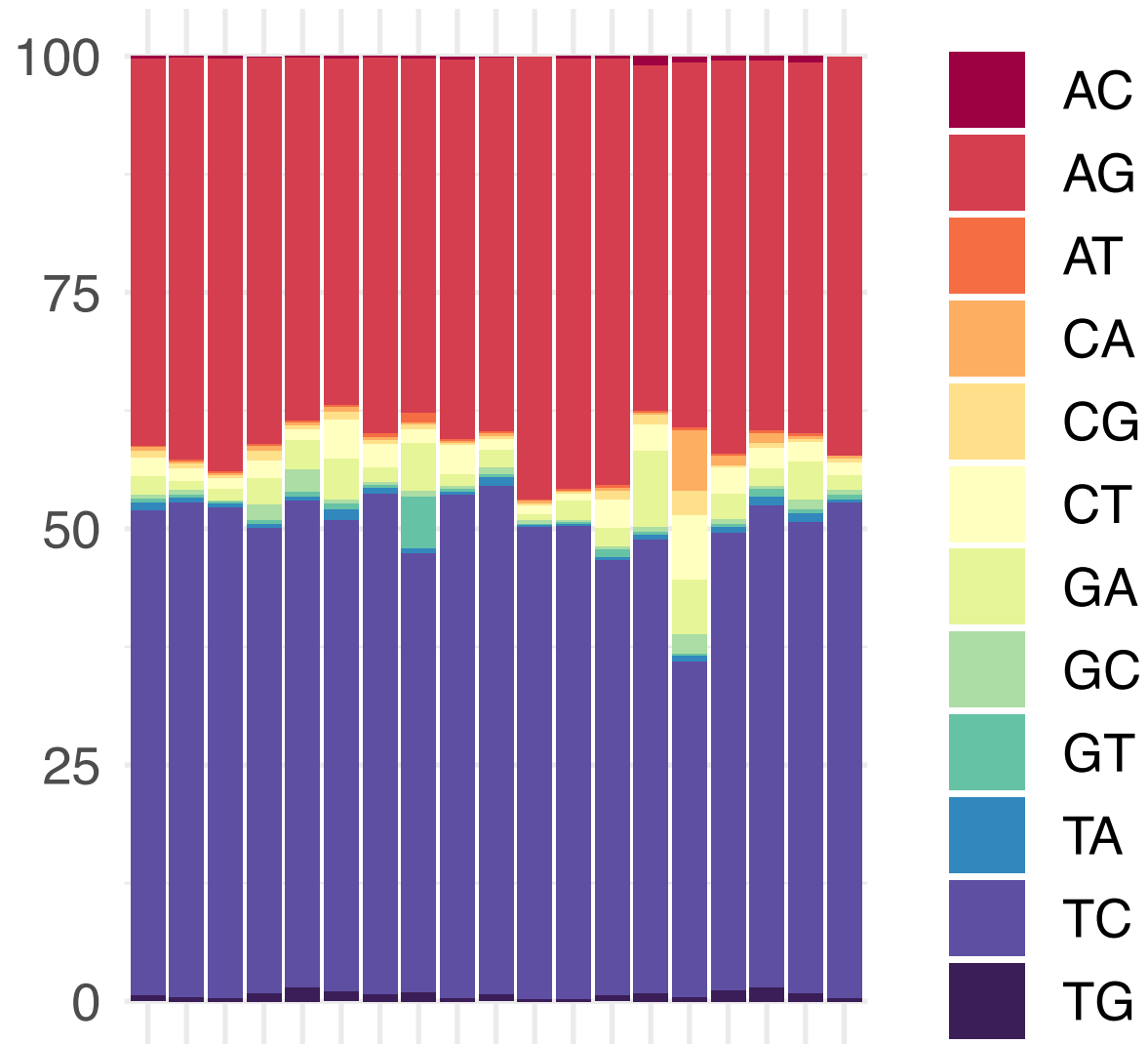

Supplement: Supplement 2 [file iovs-64-7-12_s002.pdf]

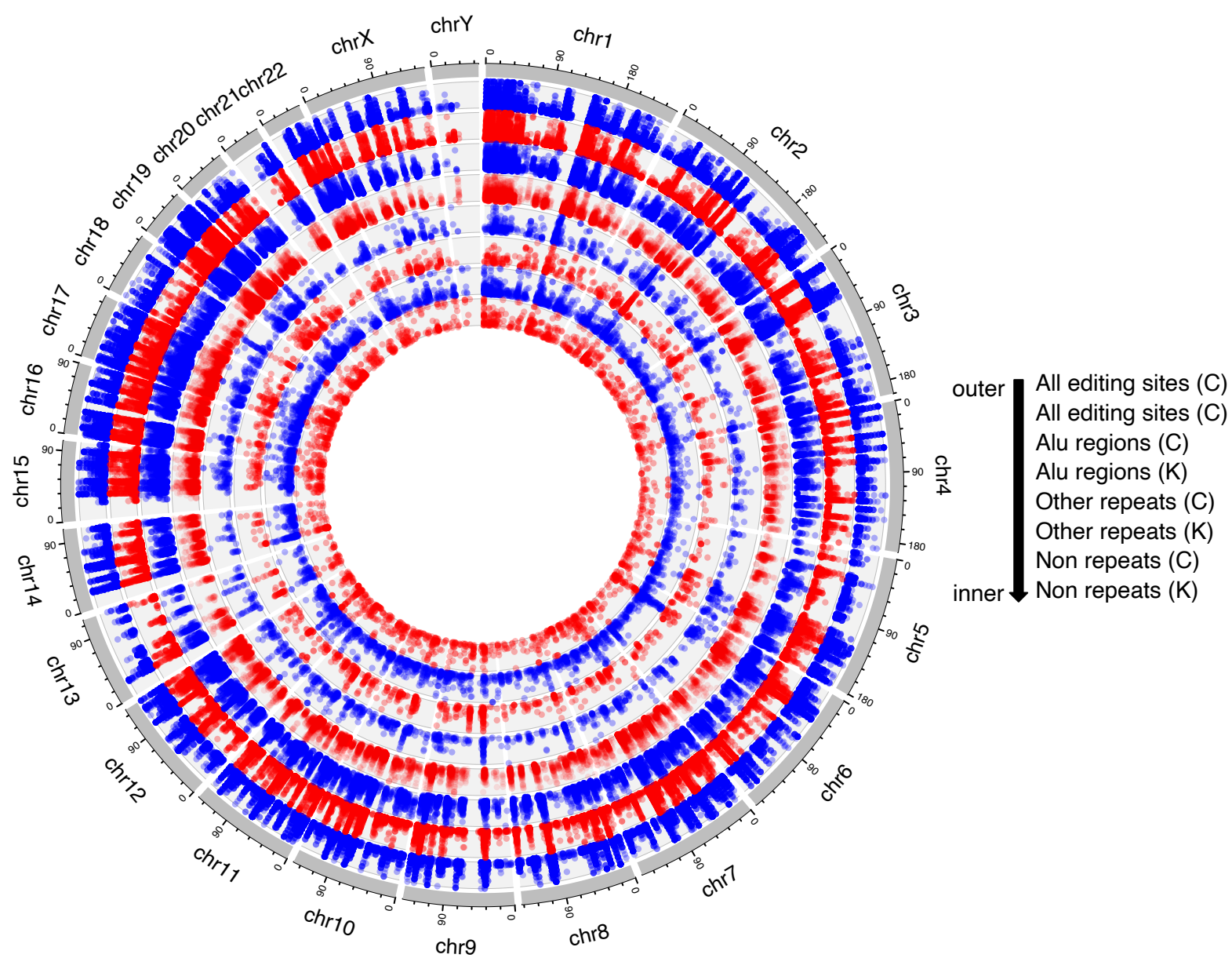

Supplement: Supplement 3 [file iovs-64-7-12_s003.pdf]

Control Keratoconus

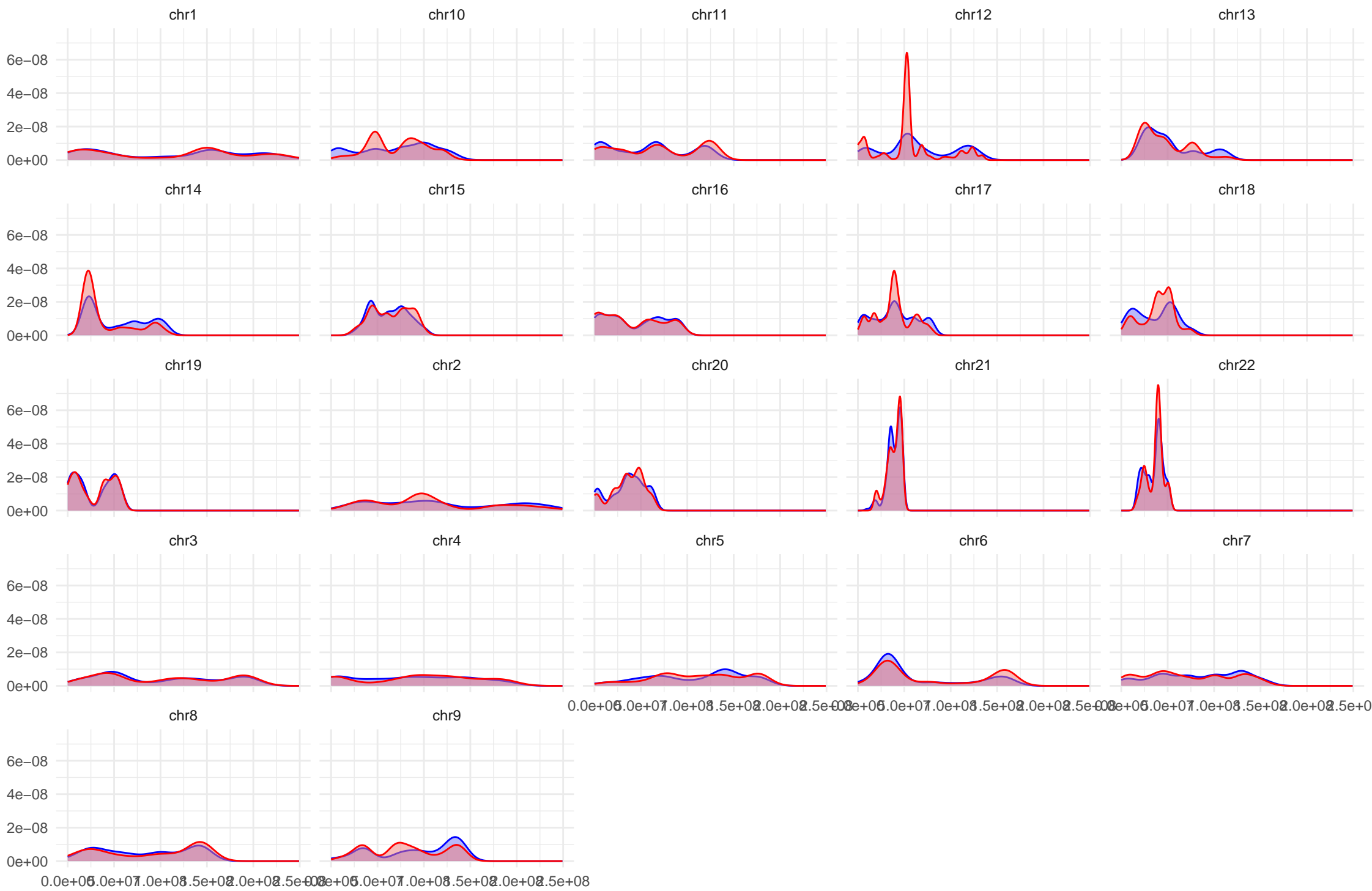

Supplement: Supplement 4 [file iovs-64-7-12_s004.pdf]

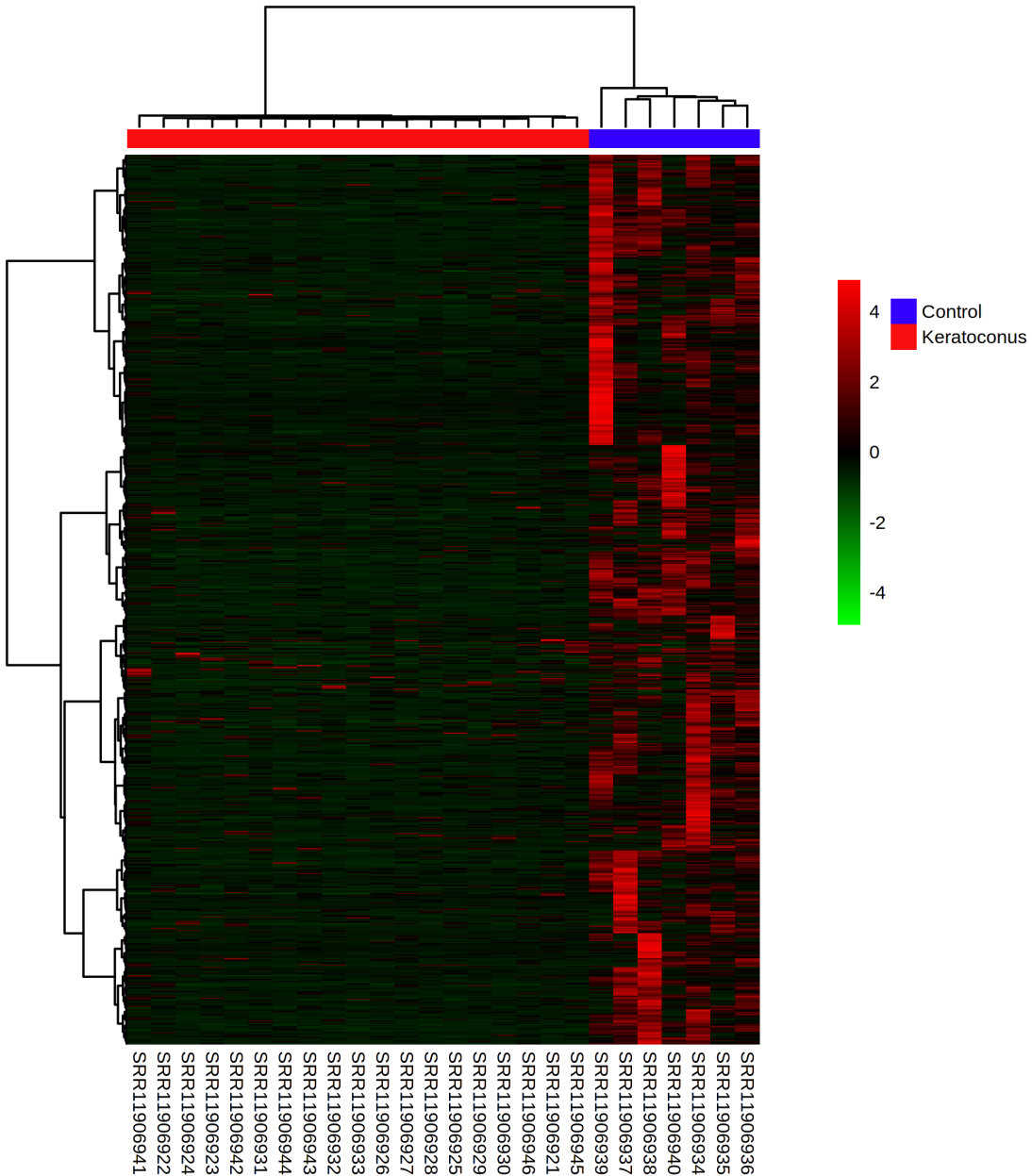

Supplement: Supplement 5 [file iovs-64-7-12_s005.pdf]
